# Decomposing the roles of perseveration and expected value representation in models of the Iowa gambling task

**DOI:** 10.3389/fpsyg.2013.00640

**Published:** 2013-09-30

**Authors:** Darrell A. Worthy, Bo Pang, Kaileigh A. Byrne

**Affiliations:** Department of Psychology, Texas A&M University, College StationTX, USA

**Keywords:** decision-making, computational modeling of decision, perseveration, expected value, iowa gambling task

## Abstract

Models of human behavior in the Iowa Gambling Task (IGT) have played a pivotal role in accounting for behavioral differences during decision-making. One critical difference between models that have been used to account for behavior in the IGT is the inclusion or exclusion of the assumption that participants tend to persevere, or stay with the same option over consecutive trials. Models that allow for this assumption include win-stay-lose-shift (WSLS) models and reinforcement learning (RL) models that include a decay learning rule where expected values for each option decay as they are chosen less often. One shortcoming of RL models that have included decay rules is that the tendency to persevere by sticking with the same option has been conflated with the tendency to select the option with the highest expected value because a single term is used to represent both of these tendencies. In the current work we isolate the tendencies to perseverate and to select the option with the highest expected value by including them as separate terms in a Value-Plus-Perseveration (VPP) RL model. Overall the VPP model provides a better fit to data from a large group of participants than models that include a single term to account for both perseveration and the representation of expected value. Simulations of each model show that the VPP model's simulated choices most closely resemble the decision-making behavior of human subjects. In addition, we also find that parameter estimates of loss aversion are more strongly correlated with performance when perseverative tendencies and expected value representations are decomposed as separate terms within the model. The results suggest that the tendency to persevere and the tendency to select the option that leads to the best net payoff are central components of decision-making behavior in the IGT. Future work should use this model to better examine decision-making behavior.

The Iowa Gambling Task (IGT) has played a critical role in the vast amount of progress that has taken place over the past two decades to develop a more complete understanding of human decision-making behavior. One of the most interesting developments in research that has utilized the IGT to examine decision-making processes has been the emergence and use of computational models to account for various aspects of behavior in the task. The Expectancy Valence (EV) model has been perhaps the most widely used model to quantitatively characterize human behavior in the task (Busemeyer and Stout, 2002; Yechiam et al., 2005, 2010; Agay et al., 2010; Hochman et al., 2010; Weller et al., 2010; Wetzels et al., 2010).

The EV model has been very useful in examining how different clinical or neuropsychological disorders affect different decision-making processes. For example, Yechiam et al. (2005) used the model to identify groups that attend more to gains than to losses (cocaine users, cannabis users, and seniors), attend more to losses than to gains (Asperger's patients), or attend to only the most recent outcomes (ventromedial prefrontal cortex patients). The EV model and other RL models have been a dominant class of models used to characterize decision-making behavior in numerous studies (Sutton and Barto, 1998; Worthy et al., 2007; Gureckis and Love, 2009a,b). The basic assumptions underpinning the EV model, and other related RL models, is that outcomes of past decisions are integrated to determine expected reward values for each option, and that decision-makers select options with higher expected rewards with greater probability than options with lower expected rewards.

Although the EV model has been widely used, recent work has found that other models can provide a better account of behavior in the task. One such model is another RL model called the Prospect Valence Learning (PVL) model (Ahn et al., 2008, 2011). One advantage of the PVL model is that it assumes that the weight people give to gains and losses follows the assumptions of Prospect Theory (Kahneman and Tversky, 1979). An additional assumption of the best-fitting version of the PVL model is the assumption that expected values for each option decay over trials. The EV model has primarily utilized a Delta learning rule that is also known as a Rescorla-Wagner rule (Rescorla and Wagner, 1972; Sutton and Barto, 1998; Yechiam and Busemeyer, 2005). This rule assumes that the expected values for each option are recency-weighted averages of the rewards received on each trial. These expected values remain unchanged until an option is chosen on a different trial. In contrast, a Decay learning rule assumes that expected values for each option decay on each trial (Erev and Roth, 1998).

The Decay rule effectively assumes that options that are not chosen will decline in expected value. Consequently, an option will become increasingly more likely to be selected the more frequently it has been selected in the recent past because its value, relative to the value of all other options, will increase due to the decaying values of the unchosen options. Thus, models that assume a Decay rule allow for the assumption that participants will *persevere* by repeatedly selecting the same option.

Another model that allows for the same assumption of perseveration, and has also provided good fits to IGT data, is a win-stay-lose-shift (WSLS) model (Worthy et al., 2012, 2013). The WSLS model assumes that participants stay (persevere) with a certain probability by picking the same option if the net reward on the previous trial was greater than zero (a “win” trial), and switch with a certain probability by picking a different option if the net reward on the previous trial was less than zero (a “lose” trial). The win-stay and lose-shift probabilities are free parameters in the model, allowing the model to account for perseverative behavior in which people sample an option repeatedly over several trials.

The WSLS and PVL models both provide better fits to data than the EV model that utilizes a Delta learning rule (Worthy et al., 2013). However, the PVL and WSLS models that have been utilized to date have a critical shortcoming in how they represent the expected values for each option. The WSLS model assumes that participants do not use any information about the relative value of each option and respond only based on whether the previous trial had a positive or negative outcome. This is a questionable assumption, at best, as it is very likely that participants give at least some consideration to the rewards they expect to receive when they select each option. The PVL model is structured so that expected reward values for each option are compared against each other to determine choice. However, the tendency to select the option with the highest expected value is conflated with the tendency to persevere by picking the same option over consecutive trials because the model uses a single value to represent both of these tendencies.

In the current work we decompose the tendency to persevere and the tendency to select options based on their reward value by developing a Value-Plus-Perseveration (VPP) model that includes separate terms to represent perseveration and expected value. Similar approaches have been utilized in other decision-making tasks by adding autocorrelation terms that are identical in form to the Decay rule (Lau and Glimcher, 2005; Schönberg et al., 2007; Kovach et al., 2012). The assumption underlying this modeling approach is that tendencies for perseveration and maximization of expected value are two fundamental, but separate aspects of decision-making. As we will show, fits of the VPP model provide a better account to data from human participants. The parameter estimates are also more informative in that parameters measuring important aspects of behavior that are assessed using the IGT, like loss aversion (Weller et al., 2010), are more strongly associated with behavior when expected value representation is decomposed from the tendency to persevere. Additionally, simulations from the VPP model are also more closely aligned with participants' data when including the number of trials that participants switched to a different option over the course of the task. Models that don't include a perseveration component tend to over-predict switch trials or under-predict perseverative behavior, while models that conflate perseveration and maximization of expected value tend to under-predict switch trials.

In the following sections we first present the models we fit to our data. We then present the methods for our experiment where participants performed the original version of the IGT (Bechara et al., 1994), followed by the behavioral and modeling results which include a comparison of each model's simulated performance and the performance of our participants. We conclude by discussing the implications of our results and by suggesting that this approach, or similar modeling approaches, be utilized to examine IGT behavior in different participant groups.

## Model descriptions

The RL models that have been fit previously to IGT data have had three components: a utility function, a value-updating rule, and an action-selection rule. The first component, the utility function, determines the degree to which gains are weighed relative to losses. The EV utility function assumes that gains and losses are simply differentially weighted. After a choice is made and feedback [points gained, win(*t*), and lost, loss(t)] is presented, the utility *u*(*t*) for the choice made on trial *t* is given by:

(1) u(t)=w · win(t)−(1−w) · loss(t)

*w* (0 ≤ *w* ≤ 1) represents the degree to which participants weigh gains vs. losses. Values greater than 0.50 indicate greater weight for gains than losses.

The Prospect Valence utility function assumes that the evaluation of each outcome follows the utility function derived from Prospect Theory (Kahneman and Tversky, 1979; Ahn et al., 2008), which has diminishing sensitivity to increases in magnitude, and different sensitivity to losses vs. gains. The utility, *u(t)*, on trial *t*, of each net outcome, *x(t)*, is:

(2) $$u(t)=\{\begin{array}{ll} x{\left(t\right)}^{\alpha } & if\;x(t)\geq 0\\ -\lambda {\left|x(t)\right|}^{\alpha } & if\;x(t)<0 \end{array}$$

Here α is a shape parameter (0 < α < 1) that governs the shape of the utility function, and λ is a loss aversion parameter (0 < *λ* < 5) that determines the sensitivity of losses compared to gains. If an individual has a value of λ greater than 1, it indicates that the individual is more sensitive to losses than gains, and a value less than 1 indicates greater sensitivity to gains than to losses.

The second component, the value-updating rule, determines how the utility *u(t)* is used to update expected values or expectancies *E_j_(t)* for the chosen option, *i*, on trial *t*. The Delta rule assumes that Expectancies are recency-weighted averages of the rewards received for each option:

(3) $$E_{i}(t)=E_{i}(t-1)+\phi \;\cdot \;[u(t)-E_{i}(t-1)]$$

The recency parameter (0 ≤ *ϕ* ≤ 1) describes the weight given to recent outcomes in updating expectancies. Higher values indicate a greater weight to recent outcomes.

The Decay rule (Erev and Roth, 1998) assumes that Expectancies of all decks decay, or are discounted, over time, and then the Expectancy of the chosen deck is added to the current outcome utility:

(4) $$E_{i}(t)=A\;\cdot \;E_{i}(t-1)+\delta_{i}(t)\;\cdot \;u(t)$$

The decay parameter *A* (0 ≤ *A* ≤ 1) determines how much the past expectancy is discounted. δ_*j*_(*t*) is a dummy variable that is 1 if deck *j* is chosen and 0 otherwise.

The third component, the action-selection rule, is a Softmax rule that determines the predicted probability that deck *j* will be chosen on trial *t*, Pr[*G_j_(t)*], is calculated using a Softmax rule (Sutton and Barto, 1998):

(5) $$Pr(G_{j}(t))=\frac{e^{\left[\theta (t)\;\cdot \;E_{j}(t)\right]}}{\sum_{j=1}^{4}e^{\left[\theta (t)\;\cdot \;E_{j}(t)\right]}}$$

In the present work we utilize a trial-independent action-selection^1^ rule for all the RL models fit to the data:

(6) $$\theta (t)=3^{c}-1$$

where *c* (0 ≤ *c* ≤ 5) is the response consistency or exploitation parameter. Larger values of *c* indicate a greater tendency to select options with higher expected values, while smaller values indicate a greater tendency explore options with lower expected values.

We first fit a total of four single-term RL models that were derived from the factorial combination of two utility functions (PVL and EV) and two value-updating rules (Decay and Delta rules). As will be described in greater detail below, we found that the PVL Delta Rule model provided a better fit to the data than the EV Delta Rule model. Given the better fit of the PVL Delta rule model, we used the PVL utility function and a Delta rule to determine the expected reward value on each trial for the two-term VPP model. The PVL utility function has also been found to outperform the EV utility function in other recent work (Ahn et al., 2008). Thus, in the VPP model the values for the first term, the expected values or expectancies [*E_j_(t)*] for each *j* choice, were determined based on Equations (2) and (3) above.

The second term, the perseveration [*P_j_*(*t*)] strengths for each *j* option were determined by a more general form of the Decay rule that has been used to model perseveration or autocorrelation among choices in recent work (Schönberg et al., 2007; Kovach et al., 2012). The perseveration term for chosen option *i*, on trial *t*, differed based on whether the net outcome, *x(t)*, was positive or negative:

(7) $$P_{i}(t)=\{\begin{array}{ll} k\;\cdot \;P_{i}(t-1)+\varepsilon_{\text{pos}} & if\;x(t)\geq 0\\ k\;\cdot \;P_{i}(t-1)+\varepsilon_{\text{neg}} & if\;x(t)<0 \end{array}$$

Here *k* (0 ≤ *k* ≤ 1) is a decay parameter similar to *A* in Equation (4) above. The tendency to perseverate or switch is incremented each time an option is chosen by ε_pos_ and ε_neg_ which we allowed to vary between −1 and 1. Positive values indicate a tendency to persevere by picking the same option on succeeding trials, while negative values indicate a tendency to switch.

The overall value of each option was determined by taking a weighted average of the two terms in the model, the expected value and the perseveration strength of each *j* option:

(8) $$V_{j}(t)=w_{E_{j}}\;\cdot \;E_{j}(t)+(1-w_{E_{j}})\;\cdot \;P_{j}(t)$$

where *w_E_j__* (0 ≤ *w_E_j__* ≤ 1) is the weight given to the expected value for each option. Values greater than 0.5 indicate greater weight based on the expected value of each option, and values less than 0.5 indicate greater weight based on the perseverative strength of each option.

These values *V_j_*(*t*) were entered into a Softmax rule to determine the probability of selecting each option, *j*, on each trial, *t*:

(9) $$Pr(G_{j}(t))=\frac{e^{\left[\theta (t)\;\cdot \;V_{j}(t)\right]}}{\sum_{j=1}^{4}e^{\left[\theta (t)\;\cdot \;V_{j}(t)\right]}}$$

where θ(*t*) was determined based on Equation (6) above.

In addition to fitting the RL models described above we also fit a WSLS model and a Baseline model. The WSLS model we used in the present work has two free parameters and is identical to the model used in prior work from our lab (Worthy et al., 2013). The first parameter represents the probability of staying with the same option on the next trial if the net gain received on the current trial is equal to or greater than zero:

(10) $$P(G_{j}(t)|{\text{choice}}_{t-1}=G_{j}\;\&\;r(t-1)\geq 0)=P(\text{stay|win})$$

In Equation (10) *r* represents the net payoff received on a given trial where any loss is subtracted from the gain received. The probability of switching to another option following a win trial is 1−P(stay | win). To determine a probability of selecting each of the other three options we divide this probability by three, so that the probabilities for selecting each of the four options sum to one.

The second parameter represents the probability of shifting to the other option on the next trial if the reward received on the current trial is less than zero:

(11) $$P(G_{j},\;(t)|{\text{choice}}_{t-1}=G_{j}\;\&\;r(t-1)<0)=P(\text{shift|loss})$$

This probability is divided by three and assigned to each of the other three options. The probability of staying with an option following a “loss” is 1−*P*(shift|loss).

Finally, the Baseline model assumes fixed choice probabilities (Yechiam and Busemeyer, 2005; Gureckis and Love, 2009a; Worthy and Maddox, 2012). The Baseline model has three free parameters that represent the probability of selecting Deck A, B, or C (the probability of selecting the Deck D is 1 minus the sum of the three other probabilities).

The right column of Table 2 lists the equations used for each model.

## Method

### Participants

Thirty-five (22 females) undergraduate students from Texas A&M University participated for partial fulfillment of a course requirement.

### Materials and procedure

Participants performed the experiment on PCs using Matlab software with Psychtoolbox (version 2.5). Participants were given the following instructions:

In this study we are interested in how people use information to make decisions.

You will repeatedly select from one of four decks of cards, and you could gain or lose points on each draw. You will be given 2000 points to start and your goal is to try to finish with at least 2500 points.

Each time you draw, the card you picked will be turned over and the number of points you gained and lost will be displayed.

You will press the ‘Z’, ‘W’, ‘P’, and ‘?/’ keys to draw from each deck.

Just do your best to maximize your gains and minimize your losses so you can finish with at least 2500 points.

Press any key to begin.

On each of 100 trials four decks appeared on the screen and participants selected one deck. Upon each selection the computer screen displayed the card choice, reward, penalty and net gain beneath the card decks. The total score was displayed on a score bar at the bottom of the screen. The task was self-paced, and participants were unaware of how many card draws they would receive. The schedule of rewards and penalties was identical to those used in the original IGT (Table 1; Bechara et al., 1994).

**Table 1:** **Reward schedule for the IGT**.

	**Deck A**	**Deck B**	**Deck C**	**Deck D**
**DRAW FROM DECK**				
1	100	100	50	50
2	100	100	50	50
3	100, **−150**	100	50, **−50**	50
4	100	100	50	50
5	100, **−300**	100	50, **−50**	50
6	100	100	50	50
7	100, **−200**	100	50, **−50**	50
8	100	100	50	50
9	100, **−250**	100, **−1250**	50, **−50**	50
10	100, **−350**	100	50, **−50**	50, **−250**
Cumulative payoff	−250	−250	250	250

*See Bechara et al. (1994) for the full table which lists payoffs for the first 40 cards drawn from each deck. In the present task the sequence was repeated for cards 41–80 and 81–100 so that a participant could potentially select the same deck on all 100 draws. Bold values indicate amount lost on each trial*.

## Results

**Figure 1:**
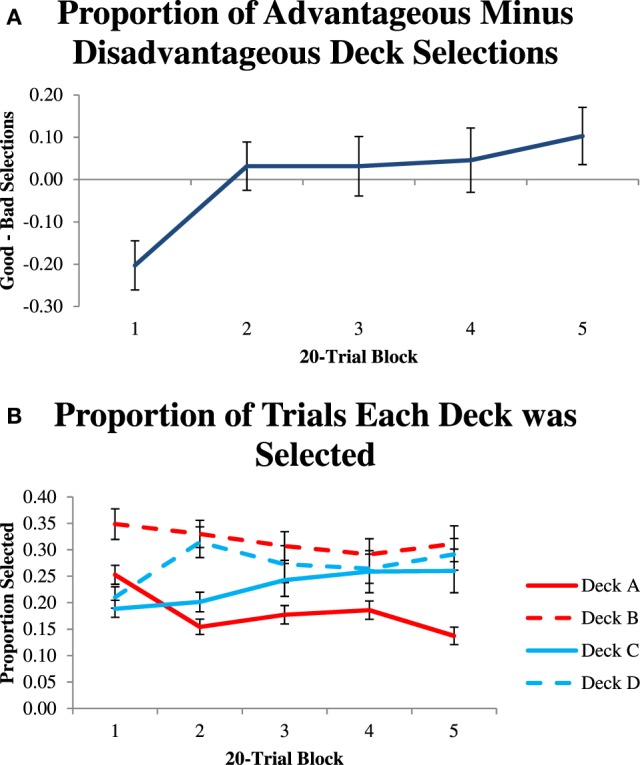
**(A)** Proportion of advantageous minus disadvantageous deck selections in 20-trial blocks. **(B)** Proportion of trials that each deck was selected in 20-trial blocks.

We first computed a performance measure that was the proportion of trials when participants selected the good decks minus the proportion of trials that they selected the bad decks. Figure 1 shows these performance values over five 20-trial blocks. A repeated measures ANOVA revealed a significant effect of block, *F*_(4)_ = 5.46, *p* < 0.001, partial η^2^ = 0.14, which suggests that participants learned to select the advantageous decks more over the course of the experiment.

### Modeling results

Models were fit individually to each participant's data by maximizing the log-likelihood for each model's prediction on each trial. We used Akaike's Information Criterion (AIC) (Akaike, 1974) and the Bayesian Information Criterion (BIC) (Schwarz, 1978) to examine the fit of the each model relative to the fit of the Baseline model. AIC penalizes models with more free parameters. For each model, *i*, AIC_*i*_ is defined as:

(12) $${\text{AIC}}_{i}=-2\text{log}L_{i}+2V_{i}$$

where *L_i_* is the maximum likelihood for model *i*, and *V_i_* is the number of free parameters in the model. BIC is defined as:

(13) $${\text{BIC}}_{i}=-2\text{log}L_{i}+V_{i}\text{log}(n)$$

where *n* is the number of trials. Smaller AIC and BIC values indicate a better fit to the data. Average AIC and BIC values for each single-term model are listed at the top of Table 2. The fits of the two Decay rule models were very similar, and better than the fits of the Delta rule models. Of the two Delta rule models, the model with a PVL utility function provided a much better fit than the model with an EV utility function. Overall, the VPP model provided the best fit to the data, based on both AIC and BIC.

**Table 2:** **Average AIC values and average Akaike weights for each model**.

	**Equations used**	**AIC**	**BIC**
EV delta	1, 3, 5–6	264.99 (26.97)	272.81 (26.97)
PVL delta	2–3, 5–6	246.62 (48.92)	260.71 (48.92)
EV decay	1, 4–6	232.94 (47.78)	240.76 (47.78)
PVL decay	2, 4–6	233.86 (54.76)	244.28 (54.76)
VPP model	2–3, 6, 8–10	211.75 (48.15)	232.60 (48.15)
WSLS model	11–12	231.76 (47.95)	236.97 (47.95)
Baseline model	NA	261.42 (31.08)	269.24 (31.08)

*Standard deviations are listed in parentheses*.

#### Simulations

Next we performed simulations for each learning model (all models except the Baseline model) to examine the proportion of trials that each model selected each option. We also examined the proportion of trials that each model switched to a different option, which is an index of the general propensity to persevere or switch. We used the parameter values that best fit our participants' data for the simulated data sets. For each model we generated 1000 data sets using parameter combinations that were sampled with replacement from the best-fitting parameter combinations for participants in our Experiment. Thus, for the EV Delta rule model we randomly sampled a combination of *w*, *ϕ*, and *c* that provided the best fit to one participant's data and used those parameter values to perform one simulation of the task. We generated 1000 simulated data sets in this manner, and performed the same simulation procedure with each learning model. This is the same approach that we've followed in recent work from our lab (Worthy et al., 2012, 2013).

**Figure 2:**
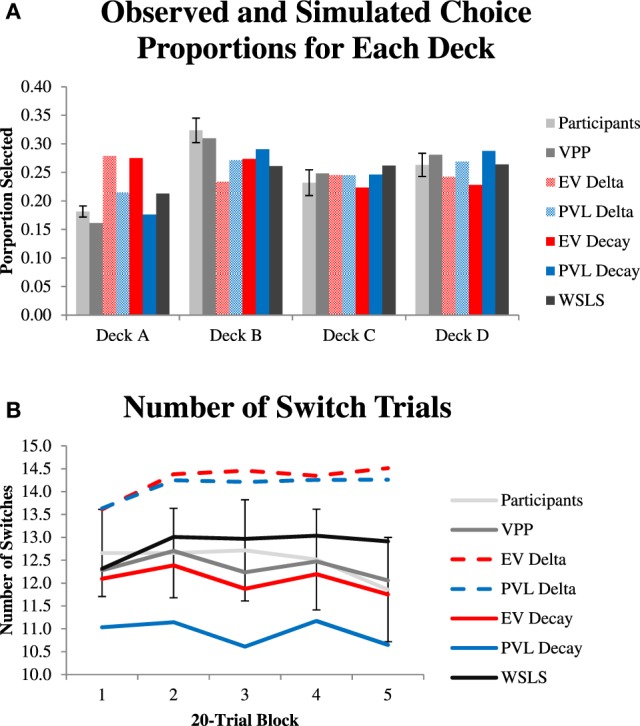
**(A)** Observed and simulated choices of each deck. Simulations randomly sampled with replacement sets of the best-fitting parameters for participants for each model. **(B)** Number of “switch” trials where participants selected a different deck than the one selected on the previous trial in 20-trial blocks.

Figure 2A shows the average proportion of times participants and each model selected each option throughout the task. The VPP model's simulated choices most closely mirror the choices made by participants, although it slightly under-predicts Deck A and B selections and slightly over-predicts Deck C and D selections. Figure 2B shows the proportion of switch trials by participants and by each model in 20-trial blocks of the task. Across all trials, the simulated switch trials for the VPP model are nearly equivalent to the average number of switch trials for participants, and are equivalent if rounded to the nearest whole number (62–62.4 for participants and 61.75 for the VPP model's simulations). Relative to the average switches made by participants, the two single-term Delta rule models, which do not have mechanisms to allow for perseveration, switched more often during their simulations. In contrast, the two single-term Decay rule models, which do have mechanisms to allow for perseveration, switched less often during their simulations. Thus, the Delta rule models under-predicted perseverative behavior, and the Decay rule models slightly over-predicted perseverative behavior.

#### Parameter estimates

Table 3 lists the average best fitting parameter values for each model along with the correlations between each parameter and performance over the entire task (proportion of Advantageous minus Disadvantageous deck selections). Of the four single-term RL models, the only parameter that was significantly associated with performance was the learning rate parameter (*ϕ*) for the PVL Delta rule model. Lower values of this parameter were associated with better performance. This could suggest that less attention to the most recent outcomes, and more attention to outcomes received over longer periods of time, may have led to better estimates of each option's expected value.

**Table 3:** **Average parameter estimates from maximum likelihood fits and association with performance for each parameter**.

	**Average**	**Correlation with performance**
**EV DELTA**		
*w*	0.58 (0.39)	−0.32
*ϕ*	0.62 (0.41)	−0.14
*c*	0.64 (0.38)	−0.13
**PVL DELTA**		
α	0.48 (0.40)	−0.27
λ	1.12 (1.91)	0.31
*ϕ*	0.61 (0.37)	−0.34^*^
*c*	1.13 (1.27)	0.31
**EV DECAY**		
*w*	0.44 (0.43)	−0.04
*A*	0.43 (0.30)	0.09
*c*	0.82 (0.25)	0.24
**PVL DECAY**		
α	0.43 (0.42)	−0.29
λ	2.56 (2.37)	0.05
*A*	0.54 (0.31)	0.05
*c*	0.47 (0.06)	0.09
**VPP MODEL**		
α	0.58 (0.39)	−0.14
λ	1.15 (1.97)	0.60^***^
*ϕ*	0.39 (0.37)	−0.23
ε_pos_	0.01 (0.66)	−0.12
ε_neg_	−0.31 (0.68)	0.25
*K*	0.47 (0.32)	0.19
*w*_*E*_*j*__	0.49 (0.34)	−0.02
*c*	3.08 (2.54)	−0.34^*^
**WSLS MODEL**		
*P*(stay|win)	0.40 (0.30)	0.09
*P*(shift|loss)	0.80 (24)	−0.37^*^

*Standard deviations are listed in parentheses*.

* *Significant at p < 0.05 level*,

*** *Significant at p < 0.001 level*.

Additionally, the VPP model's estimated exploitation parameter values (c) were also positively associated with performance. We also observed a significant positive association between the WSLS models estimated lose-shift *P*(shift|loss) parameter values and performance, which suggests that participants performed better if they were more likely to select a different option following a net loss.

**Figure 3:**
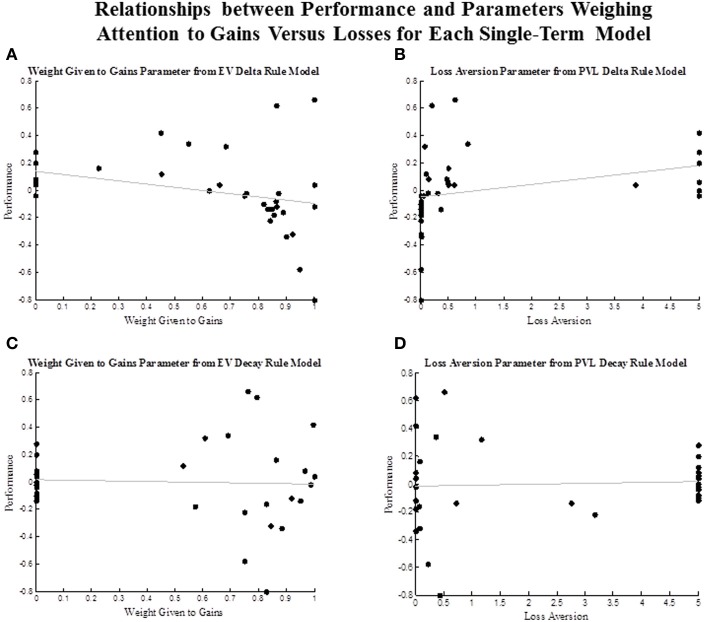
**Scatterplot of the association between performance and parameter estimates that weigh the attention given to gains vs. losses. (A)** association between performance and the attention to gains ***(w)*** parameter from the EV Decay Rule model. **(B)** association between performance and the loss aversion parameter from the PVL Delta Rule model. **(C)** association between performance and the attention to gains ***(w)*** parameter from the EV Decay Rule model. **(D)** association between performance and the loss aversion parameter from the PVL Decay Rule model.

Recent work suggests that greater attention to losses than to gains is beneficial in the IGT (Weller et al., 2010). Therefore, we were interested in examining how estimates of parameters that accounted for attention to gains vs. losses were associated with performance in the task. Figure 3 plots these associations for each single term model. The attention to gains parameter (*w*) in the EV Delta rule model was negatively associated with performance, and the loss aversion parameter (*λ*) from the PVL Delta rule model was positively associated with performance. Although, the associations between these parameters and performance only approached significance, estimated values of these same parameters had basically no relationship with performance in the EV Decay (*r* = −0.04 for *w*) and PVL Decay models (*λ* = 0.05, where the tendency to select options based on their expected values is conflated with the tendency to persevere.

**Figure 4:**
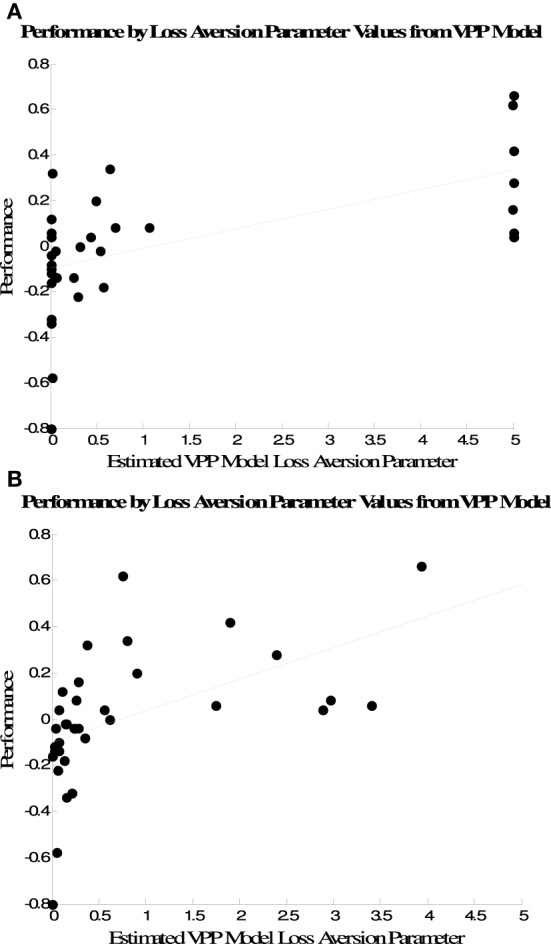
**Association between performance and loss aversion parameter estimates from the VPP model when parameters are estimated via (A) maximum likelihood and (B) Bayesian hierarchical estimation**.

There was a strong association between performance and estimated loss aversion (*λ* parameter values from the VPP model (Figure 4A). One point to note is that many participants' data were best fit by extreme values along the bounds for these parameters from both the VPP and the single-term models. Recent work has demonstrated that a potential anomaly of the estimating parameters for individual participants via maximum likelihood is that many estimates will fall on the bounds of the parameter space (Wetzels et al., 2010; Ahn et al., 2011). Thus, it is difficult to determine whether the extremely low or extremely high loss aversion parameter values indicated exclusive attention to gains or losses by some subjects, or whether those values were due to problems with estimating parameters for individual subjects via maximum likelihood.

To address this issue we estimated the VPP model's parameters using a Bayesian hierarchical procedure that has recently been used to estimate parameters from the EV Delta rule model for IGT data (Wetzels et al., 2010). While the maximum likelihood approach provides a single best-fitting set of parameters for each subject, the Bayesian hierarchical approach yields posterior distributions for each parameter that quantify the uncertainty about each parameter, given the data. Posterior distributions were estimated based on a total of 30,000 MCMC samples from three chains, after 1000 burn-in samples. Figure 4B plots the association between performance and the mode of each subject's posterior distribution for the loss aversion parameter from the VPP model. Similar to the estimates from maximum likelihood there is a strong positive association between performance and loss aversion estimates (*r* = 0.52, *p* < 0.001). However, the modes of the posterior loss aversion parameter distributions are not at as extreme points near the bounds of the parameter space as the point estimates provided by the maximum likelihood fits. Thus, the relationship between loss aversion parameter estimates and performance is similar for both approaches, but maximum likelihood estimation is more likely to yield estimates near the bounds of the parameter space.

**Figure 5:**
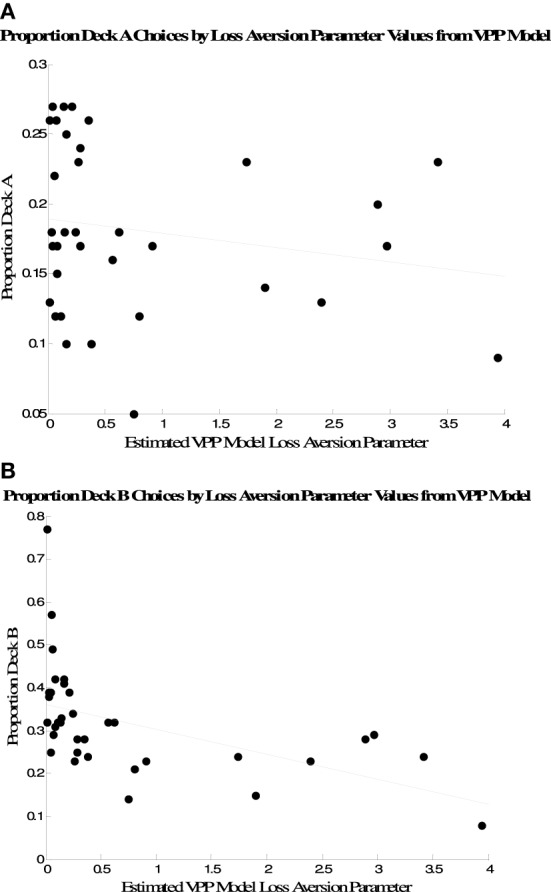
**Association between Deck A selections (A) and Deck B selections (B) and individual posterior modes of loss aversion parameter distributions from the VPP model**.

Because the measure of performance we used is only one measure among many possible ways to characterize performance on the IGT, we also examined the relationship between the mode of each subject's posterior loss aversion parameter distribution and the proportion of trials participants selected Decks A and B. These are plotted in Figure 5. There were negative associations between the VPP model's loss aversion parameters and selections of both options, but the association was only significant for Deck B selections (Deck A, *r* = −0.19, *p* > 0.10; Deck B, *r* = −0.51, *p* < 0.01).

## Discussion

We presented a VPP model that included separate terms to account for perseverative behavior and tendencies to select options based on their expected values. Overall, this model provided the best fit to the data and its simulations most closely mirrored human behavior—both the proportion of times people selected each option and how often they tended to switch to a different option. This supports our assertion that it is critical to account for both perseveration and maximization of expected value in models of human decision-making behavior in tasks like the IGT, and it is also critical to ensure that these tendencies are decomposed in the model. People vary in both their tendency to select more advantageous options and in their tendency to “stay” or “switch” on successive trials.

There was a very strong relationship between the VPP model's best-fitting loss aversion parameter values and performance in the IGT using both maximum likelihood and Bayesian hierarchical approaches to obtain individual parameter estimates. This supports recent work that suggests that loss aversion is a critical component, perhaps the most critical component, of successful performance in the IGT (Weller et al., 2010). The role of loss aversion is intuitively obvious in that the distinguishing feature between the advantageous and disadvantageous decks is that, over time, the latter provide net losses, while the former provide net gains. The relationships between estimated loss aversion parameter values and performance sharply differed based on the learning rule that was used. Parameters that accounted for attention to losses vs. gains from the single-term Delta rule models both showed associations with performance (albeit weak ones) that suggest that enhanced attention to losses improves IGT performance. In contrast, there was basically no relationship between parameter estimates of attention to losses and performance for the single-term Decay rule models. This is an important point because these models differ based on their assumptions of how important loss aversion is for successful performance in the task. We propose that the null relationship between loss aversion parameter estimates and performance for the Decay rule models is due to the conflation between representations for expected value maximization and perseverative behavior.

Additionally, it is important to note that loss aversion and attention to gains parameter estimates from all the models we fit via maximum likelihood estimation were not normally distributed. Many data sets were best fit by extreme values for these parameters which may be an anomaly that comes from to estimating parameter using maximum likelihood. Bayesian hierarchical parameter estimation is an alternative method of estimating parameters that has several advantages over maximum likelihood estimation, particularly at the individual subject level (Wetzels et al., 2010).

In an elegant and very thorough analysis of model performance in the IGT and the Soochow gambling task (Lin et al., 2007; Ahn et al., 2008; Chiu et al., 2008) recently suggested that decay learning rules are better at making short-term predictions, like which option would be chosen on the next trial, while Delta rule models are better at making long-term predictions, like an entire sequence of choices. For example, a model that included a Delta rule may provide a poorer fit to a participant's data, but parameter estimates from a Delta rule model would be better at predicting behavior for the same individual in another decision-making task. We propose that the advantage in short-term prediction for Decay rule models is due to their ability to account for perseverative behavior, and the advantage in long-term prediction for Delta rule models is due to their ability to better account for things like loss aversive tendencies, which affect how participants value options. While we did not use the generalization criterion method (Busemeyer and Wang, 2000) of using parameter estimates from fits to data from one task to predict subsequent behavior in another task in the current work, we predict that isolating perseveration and expected value representation in learning models, like the VPP model we presented here, would improve both short- and long-term predictions. Indeed prior work has found that the EV model, which does not conflate expected value representation with perseveration, was more successful in the generalization criterion method than in fits to a single dataset (Yechiam and Busemeyer, 2008; Kudryavtsev and Pavlodsky, 2012). Although our study did not utilize the generalization criterion method we would predict that the VPP model would perform well in predicting behavior on subsequent tasks.

The development of the IGT 20 years ago has led to excellent cross-cutting research across various sub-disciplines in psychological science. Decision-making is a critical component of everyday behavior, and the IGT has been the most frequently used experimental task designed to assess poor decision-making, particularly among patient groups (Bechara et al., 2001; Boeka and Lokken, 2006; Lakey et al., 2007). However, the IGT is also a complex task and basic analyses of performance in the task, like the proportion of advantageous vs. disadvantageous choices, do not provide a full account of decision-making behavior. We argue that model-fitting is a critical tool that can be applied to IGT data to allow for a more complex examination of how decision-making varies among groups and individuals. We found a strong link between loss aversion and performance in the IGT. However, other approaches, like the ones used by Yechiam et al. (2005), can be used to compare parameter estimates between different patient populations to identify how different groups attend to recent outcomes, attend to gains vs. losses, select options with greater expected values or tend to persevere vs. frequently switch options. It is our view that the biggest insights into decision-making behavior in tasks like the IGT will continue to come from approaches that include both behavioral and computational analyses of data that are collected from a wide variety of participants and groups.

### Conflict of interest statement

The authors declare that the research was conducted in the absence of any commercial or financial relationships that could be construed as a potential conflict of interest.

## Footnotes

^1^A trial-dependent rule has also been applied to models that have been fit to IGT data (Yechiam and Busemeyer, 2005). We found that the pattern between the relative fit of each model we present was the same regardless of which action selection rule was used and that the trial-independent rule fit best in most cases. Therefore, for simplicity we only use the trial-independent rule in the present work.
